# TIGAR coordinates senescence-associated secretory phenotype via lysosome repositioning and α-tubulin deacetylation

**DOI:** 10.1038/s12276-024-01362-4

**Published:** 2024-12-04

**Authors:** Hae Yun Nam, Seung-Ho Park, Geun-Hee Lee, Eun-Young Kim, SangEun Lee, Hyo Won Chang, Eun-Ju Chang, Kyung-Chul Choi, Seong Who Kim

**Affiliations:** 1 Department of Biochemistry and Molecular Biology, Brain Korea 21 project, Asan Medical Center, University of Ulsan College of Medicine, Seoul, 05505 South Korea; 2 Department Hematology and Medical Oncology, Whinship Cancer Institute of Emory University, Atlanta, GA 30322 USA

**Keywords:** Cell growth, Biomarkers

## Abstract

TP53-induced glycolysis and apoptosis regulator (TIGAR) regulates redox homeostasis and provides the intermediates necessary for cell growth by reducing the glycolytic rate. During cellular senescence, cells undergo metabolic rewiring towards the glycolytic pathway, along with the development of the senescence-associated secretory phenotype (SASP), also known as the secretome. We observed that TIGAR expression increased during replicative senescence following the in vitro expansion of human mesenchymal stromal cells (MSCs) and that TIGAR knockout (KO) decreased SASP factors and triggered premature senescence with decelerated progression. Additionally, TIGAR KO impaired flexible lysosomal movement to the perinuclear region and decreased the autophagic flux of MSCs. Research on the mechanism of lysosomal movement revealed that, while native senescent MSCs presented low levels of Ac-α-tubulin (lysine 40) and increased sirtuin 2 (SIRT2) activity compared with those in growing cells, TIGAR KO-MSCs maintained Ac-α-tubulin levels and exhibited decreased SIRT2 activity despite being in a senescent state. The overexpression of SIRT2 reduced Ac-α-tubulin as a protein target of SIRT2 and induced the positioning of lysosomes at the perinuclear region, restoring the cytokine secretion of TIGAR KO-MSCs. Furthermore, TIGAR expression was positively correlated with SIRT2 activity, indicating that TIGAR affects SIRT2 activity partly by modulating the NAD^+^ level. Thus, our study demonstrated that TIGAR provides a foundation that translates the regulation of energy metabolism into lysosome positioning, affecting the secretome for senescence development. Considering the functional value of the cell-secretome in aging-related diseases, these findings suggest the feasibility of TIGAR for the regulation of secretory phenotypes.

## TIGAR modulates metabolism and lysosome positioning in senescence

Cellular senescence is a process where cells stop dividing due to stress. Researchers found that the protein TIGAR plays a role in this process, but its exact function was unclear. In this study, researchers explored how TIGAR affects cellular senescence. They used mesenchymal stromal cells from human umbilical cord blood and conducted experiments to knock out TIGAR using CRISPR-Cas9 technology. They found that knocking out TIGAR led to early onset but slower progression of senescence. TIGAR knockout cells showed changes in lysosome positioning, reduced autophagic flux, and altered secretion of cytokines. These changes were linked to the acetylation of α-tubulin, a protein that helps in cell structure and transport. The results suggest that TIGAR regulates cellular senescence by affecting lysosome positioning and autophagy through SIRT2, an enzyme that deacetylates α-tubulin.

This summary was initially drafted using artificial intelligence, then revised and fact-checked by the author.

## Introduction

Cellular senescence is an irreversible proliferation arrest mechanism that occurs in response to cellular stress. The senescence-associated secretory phenotype (SASP), described as a significant change in the secretome^1,2^, autocrinally reinforces senescent phenotypes and confers potent paracrine activities to cells, with both beneficial and deleterious effects depending on the cellular context^3^. The acquisition of senescence is closely associated with metabolic rewiring of the anabolic pathway, which accompanies the alteration in the content and activity of intracellular organelles such as lysosomes^4,5^. Thus, defining the metabolic targets for secretome regulation and uncovering their mechanisms are crucial for controlling senescence development.

TP53-induced glycolysis and apoptosis regulator (TIGAR) functions as a fructose-2,6-bisphosphatase (Fru-2,6-BP) and redirects metabolic intermediates toward the pentose phosphate pathway (PPP)^6^. Increased flux toward the PPP increases the production of nicotinamide adenine dinucleotide phosphate hydrogen (NADPH), a glutathione reductase (GR) cofactor that regenerates reactive oxygen species (ROS) scavengers and provides ribose for damaged DNA repair. Therefore, TIGAR acts as a survival factor in response to oxidative or metabolic stress signals and within the tumor microenvironment, where it has been associated with the inhibition of autophagy and apoptosis^7–9^. However, the functional roles of TIGAR expression in cellular senescence phenotypes remain unclear.

The lysosome is responsible for the degradation and recycling of unnecessary portions of the cytoplasm and for the sensing and control of cellular energy homeostasis and metabolism^10–12^. Extensive remodeling of lysosomal biogenesis and catabolic programs is commonly shared in cellular senescence at different states and from diverse origins^13^; thus, it acts as a major hallmark of senescent cells. During cellular senescence, altered lysosome programming affects autophagy and the mTOR-associated anabolic pathway^14,15^, where characteristic spatial and temporal linkages indicate that the proper position of lysosomes is a critical step.

Lysosomes move bidirectionally along microtubule tracks between the center and periphery of the cells^16^, which is mediated by the microtubule rulers, such as kinesins and dynein, toward the plus (anterograde) and minus ends (retrograde), respectively^17,18^. During cell senescence, α-tubulin acetylation on lysine 40 is associated with increased microtubule stability in renal and intestinal epithelial cells^19,20^. Moreover, α-acetylated microtubules are crucial for controlling lysosome movement from the perinuclear cloud to the plasma membrane^21^. The acetylation level of α-tubulin is modulated by the opposing activities of α-tubulin acetyltransferase (αTAT1)^22^ and deacetylases such as NAD-dependent deacetylase Sirtuin-2 (SIRT2) and histone deacetylase 6 (HDAC6)^23,24^. Taken together, understanding the specific physiological processes affected by the spatial arrangement and movement of lysosomes in cells undergoing senescence can provide insights into the complex mechanisms underlying cellular secretion and senescence signaling.

In this study, we used clues from mesenchymal stromal cells (MSCs) undergoing replicative senescence processes during in vitro expansion. Our study reveals a novel function of TIGAR in regulating SASP-associated perinuclear lysosomal distribution, hypoacetylated α-tubulin levels, and SIRT2 activity during cellular senescence.

## Materials and methods

### Cell culture

The human umbilical cord blood-derived MSCs (UCB-MSCs) were donated by MEDIPOST Co., Ltd. (Seongnam, Korea). This study was approved by the Institutional Review Board of MEDIPOST Co. Ltd. MSCs were maintained in minimum essential medium (α-MEM; Life Technologies, 11900024, Carlsbad, CA) with 5.56 mM glucose supplemented with 10% fetal bovine serum (FBS; Gibco, Life Technologies) and 1% penicillin/streptomycin (HyClone, SV30010, Logan, Utah) at 37 °C in a humidified 5% CO_2_ atmosphere. The media of the cells were changed every 2–3 days for 5 days according to a procedure specified in a previous study^25,26^. PD was continuously monitored until the cells ceased to proliferate. For interaction experiments, HEK293T cells were cultured in Dulbecco’s modified Eagle’s medium (DMEM; HyClone, SH30243.01) with 4 mM glucose supplemented with 10% FBS and 1% antibiotics.

### Plasmids and reagents

Effectene transfection reagent was purchased from QIAGEN (301427, Germany). The HA-TIGAR and Flag-SIRT2 constructs were obtained from Dr. Choi, K.C. (Ulsan University, Korea) and subcloned and inserted into the pSG5 vector to achieve transient expression in HEK293T cells. The pCDH-CMV-MCS-EF1-Puro overexpression vector was purchased from System Biosciences (CD510B-1, Palo Alto, CA). The cells stably expressing TIGAR and SIRT2 were cultured under puromycin selection (1.5 μg/mL).

Bafilomycin-A1 (Sigma, B1793, St. Louis, MO), AGK2 (Sigma, A8231), Earle’s balanced salt solution (EBSS; Gibco, 24010043), and nonessential amino acids (NEAA; Gibco, 11140035) were purchased. Reagents were prepared in dimethyl sulfoxide (DMSO) as the vehicle and treated at the final concentrations indicated in the figure legends.

### CRISPR-Cas9-mediated gene knockdown of TIGAR

Effective gene KO of TIGAR was achieved through CRISPR/Cas9-mediated genome editing. We used pLentiCRISPRv2, wherein single-guide RNA directed against a target of TIGAR and the Cas9 endonuclease were delivered to cells through a lentivirus. Three target site sequences were selected based on the best scores previously calculated for the gene. The two most effective target guide sequences determined via western blotting were used in our experiments: sgC12ofr5 TIGAR #1: CTTTGTCCTCATGAGATCAC (minus strand) and sgC12ofr5 TIGAR #2: TCTCTCCAAAATTCCATGCA (minus strand).

### Senescence-associated β-galactosidase (SA β-gal) activity

SA β-gal staining was used as a biomarker of representative senescence. The SA β-gal activity was qualitatively assessed with a β-galactosidase reporter gene staining kit (Sigma) according to the manufacturer’s instructions. The percentage of β-galactosidase-positive cells was determined by counting five fields on a phase contrast microscope (Carl Zeiss, Germany).

### Measurement of cytokine secretion

To quantify the level of secretory cytokines, cells in six-well plates (5 × 10^4^/well) were replenished with fresh complete medium and subsequently cultivated for 48 h. The culture medium was collected and clarified via centrifugation at 200 × *g* for 5 min. The amount of cytokines within the harvested medium was quantified using human IL-6 and MCP-1 ELISA kits (BioLegend, 430504 and 438804, respectively; San Diego, CA) according to the manufacturer’s protocol. The results were normalized to a cell number of 1 × 10^4^.

To investigate the change in the secretome of TIGAR-KO cells during senescence, cultured medium (3% FBS) conditioned by MSCs for 48 h, obtained from growing (nonsenescent) and senescent control cells or TIGAR-KO cells, was analyzed for 23 cytokines using a customized human premixed Multi-Analyte Kit (RND-LXSAHM-28) by LABISKOMA (Seoul, Korea).

### Quantitative real-time PCR

Gene-specific primer sets were designed using the National Center for Biotechnology Information (NCBI) primer blast or based on published studies. Total RNA was isolated using TRIzol® reagent (Invitrogen, Grand Island, NY), and 1 μg of RNA was reverse transcribed to cDNA using SuperScript III Master Mix (RT300; Enzynomics, Daejeon, Korea). Amplification was conducted with the CFX Connect^™^ Real-Time PCR Detection System (Bio-Rad, Hercules, CA) using iQ^™^ SYBR® Green Supermix (Bio-Rad) and gene-specific primers (Supplementary Table 1). *GAPDH* served as an endogenous normalization control. All expression values are expressed as the fold change, and the reaction was repeated three times.

### Western blotting

Whole-cell lysates were prepared in RIPA lysis buffer (50 mM Tris-HCl [pH 8.0], 1% NP-40, 0.5% Na-deoxycholate, 150 mM NaCl, and 0.1% SDS) containing a protease inhibitor cocktail (Thermo Scientific, 1861281, Waltham, MA) for 30 min on ice and boiled in 2× loading buffer. Protein concentrations were measured using a BCA assay kit (Thermo Scientific). Protein samples were resolved via SDS–PAGE and transferred onto a nitrocellulose membrane, which was blocked in 5% skim milk in TBST and probed with the indicated antibodies. The antibodies used are shown in Supplementary Table 2. The density of the protein signals was quantified via NIH ImageJ software.

### Immunoprecipitation

For immunoprecipitation, the cells were lysed in IP lysis buffer (0.5% NP-40, 0.5% Triton X-100, 20 mM HEPES (pH 7.4), 12.5 mM beta-glycerolphosphate, 1.5 mM MgCl_2_, 10 mM NaF, 2 mM DTT, 1 mM NaOV, 2 mM EGTA, PMSF, 150 mM NaCl, and protease inhibitor) directly followed by sonication. Clarified cell lysates were incubated with specific antibodies against HA, Flag or SIRT2 overnight at 4 °C. Immune complexes were isolated by protein A/G beads (Santa Cruz Biotechnology, Dallas, TX) for 3–5 h. Beads were washed four times with IP washing buffer and then boiled in 2× SDS sampling buffer. Immunoprecipitated proteins were subjected to western blot analyses. The antibodies used for IP are shown in Supplementary Table 2.

### Immunofluorescence

For the immunofluorescence experiments, the cells were grown on a 35-mm imaging dish (ibidi 81156, Martinsried, Germany) for 48 h before the experiments. After fixation in 4% paraformaldehyde (PFA) for 15 min and permeabilization for 10 min in 0.1% Triton X-100 in PBS, the cells were immunolabeled with the corresponding primary antibodies. Subsequent immunodetection was conducted with Alexa Fluor-conjugated antibodies (Molecular Probes, Eugene, OR). The nuclei were visualized with 4′,6-diamidino-2-phenylindole (DAPI). All images were obtained on an inverted confocal laser-scanning microscope (LSM 880; Carl Zeiss MicroImaging, Inc.) equipped with a C-Apochromat 40 ×, 1.2 W AutoCorr M27 objective. Image analysis was conducted using ImageJ or ZEN software. All the images were adjusted for brightness and contrast.

### mRFP-GFP/LC3 image analysis

The autophagy reporter plasmid ptfLC3 (encoding mRFP-GFP-MAP1LC3B; 21074) was purchased from Addgene (Cambridge, MA). According to the manufacturer’s instructions, plasmid transfection was conducted with Lipofectamine Stem Transfection Reagent (Invitrogen). At 24 h after transfection, the cells were treated with bafilomycin-A1 and/or EBSS for starvation, and >20 cells per condition were imaged on a confocal system (LSM 880; Carl Zeiss). The quantification of autophagic flux was conducted with ImageJ. Spots that were both green and red (GFP^+^/mRFP^+^) were considered autophagosomes, and those that were red (GFP^−^/mRFP^+^) were considered autolysosomes^27^.

### SIRT2 activity assay

SIRT2 activity was determined in the purified protein according to the manufacturer’s protocol (K324; BioVision Biotechnology, Milpitas, CA). The lysates of the cells were subjected to IP with an anti-SIRT2 antibody. Immunoprecipitated complexes were collected from agarose A/G beads and washed with IP washing buffer four times. The proteins bound to the beads were then used for the SIRT2 activity assay, following the instructions provided by the manufacturer.

### NAD^+^/NADH assay

The NAD^+^/NADH ratio was determined using the NAD^+^/NADH quantification kit (Abcam, ab65348, Cambridge, UK) according to the manufacturer’s instructions. Briefly, 2 × 10^5^ cells were pelleted and lysed in 400 μL of extraction buffer. Two hundred microliters of this lysate was heated at 60 °C for 30 min to decompose NAD^+^ and determine the NADH concentration. Another portion was used to determine the protein concentration for normalization.

### Isolation of macrophages

Bone marrow-derived macrophages (BMMs) were generated as previously described^28^. Mouse whole bone marrow cells were isolated by flushing the marrow space of the femora and tibiae, and the nonadherent cells were cultured for 3 days with M-CSF (30 ng/mL) to obtain BMMs. BMMs were cultured at 37 °C in a humidified atmosphere with 5% CO_2_ in α-MEM supplemented with 100 μg/mL streptomycin, 100 U/mL penicillin, and 10% FBS.

### Phagocytosis assay

After the BMMs were incubated in α-MEM supplemented with 3% FBS or the conditioned medium from the MSC cultures for 24 h, the cells were stimulated with FITC-labeled zymosan particles (Molecular Probes) for 40 min. Subsequently, zymosan was aspirated and then washed three times with PBS, and the extracellular fluorescence was quenched with 200 μL of trypan blue suspension for 1 min at room temperature. The excess trypan blue was removed by washing three times with PBS, and the mixture was then detached from the dishes with 1 mL of PBS. Phagocytosis was assayed by measuring the intensity of bioparticles engulfed in the cells, and the fluorescence of the cell lysates was fluorospectrophotometrically measured by excitation and emission at 480 and 520 nm, respectively.

### Migration assay

The migration assay was performed as described previously to evaluate the chemotactic potential of BMMs exposed to the MSC secretome^29^. Macrophages (4 × 10^4^) were plated in the upper chamber of a 5 μm pore size Transwell system (Costar, Corning, Kennebunk, ME). Conditioned medium from the MSC cultures was added to the lower chamber and incubated, while the macrophages were loaded into the upper chamber. After incubation for 24 h to enable macrophage migration, the macrophages that had not migrated were scraped from the top of the membrane with a cotton swab, and those present in the lower chamber were stained with hematoxylin and counted under a microscope.

### Statistical analysis

The collected data are expressed as the means ± SDs or means ± SEs of at least three independent experiments. All the statistical analyses were performed via SigmaPlot 10.0 or GraphPad Prism 9.5 software. The statistical significance of differences between groups was determined via two-tailed Student’s *t*-test. A *P*-value of <0.05 was considered to indicate statistical significance (*p < 0.05, **p < 0.01, ***p < 0.005). Details and the significance values can be found in the figure legends.

## Results

### MSCs increase TIGAR expression and induce senescence-associated secretory phenotypes during replicative senescence

**Fig. 1:**
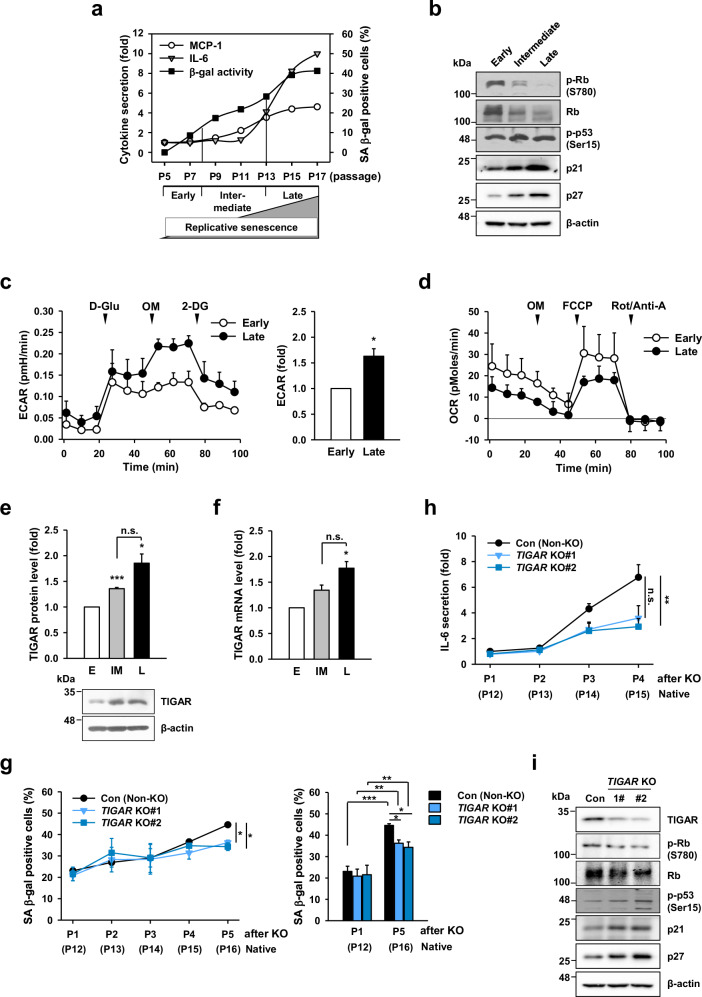
TIGAR influences senescence-associated secretory phenotype during replicative senescence of MSCs. **a** MSCs in culture were measured for the secretion of cytokines (MCP-1 and IL-6) and the percentage of cells with positive SA β-gal staining following in vitro passaging. The cytokine data are expressed as the fold change relative to the initial passage (P5). Three stages of MSC senescence were classified below the corresponding passages: early (E, P5 to P7), intermediate (IM, P8 to P12), and late (L, P13 to P17). **b** western blot analysis of cell cycle arrest markers in MSCs at each stage of replicative senescence. **c**–**f** Extracellular acidification rate (ECAR, **c**), oxygen consumption rate (OCR, **d**), and expression levels of TIGAR protein and mRNA (**e**, **f**) during the MSC senescence process. The ECAR and OCR responses were normalized to the number of cells (1.5 × 10^4^ cells). Data (**c**, **e**, **f**) are expressed as fold changes relative to the early stage. **g** The percentage of cells with positive SA β-gal staining over passages after TIGAR was knocked out via the CRISPR/Cas9 system in MSCs (P7, *n* = 4). To compare the changes in the control (non-KO) cells and TIGAR KO cells, data from the first and last passages of each cell line were converted into a bar graph. **h** IL-6 secretion over passages after TIGAR was knocked out in MSCs. The cytokine data were normalized to the number of cells (1 × 10^4^ cells) and are presented as the fold change relative to the initial passage of control cells (*n* = 4). In (**g** and **h**), the passages after KO are indicated along with the corresponding passages of native MSCs. **i** western blot analysis of the cell cycle arrest markers in TIGAR KO-MSCs (P3 after KO). Representative images of the western blots are shown in (**b** and **i**). The data are presented as the means (**a**) or means ± SDs (**c**–**h**) from three to four biological replicates. n.s.: not significant; *p < 0.05, **p < 0.01, ***p < 0.005.

We previously reported that MSCs derived from umbilical cord blood (UCB-MSCs; hereafter referred to as MSCs) lose their proliferative capacity and acquire senescence-associated (SA) phenotypes during long-term in vitro culture^30^. Based on their growth rate and profile of inflammatory cytokine secretion across intradonor differences, we classified the senescence process of MSCs into three stages: early, intermediate, and late^30^ (Fig. 1a), similar to time-ordered cellular senescence described in a previous report^31^. The development of senescence in MSCs was verified by SA β-galactosidase (SA β-gal) activity and the secretion of cytokines and cell cycle checkpoints (pRb, p53, p21, and p27; Fig. 1a, b). We observed that after an initial increase in SA β-gal activity around passages 7–9, the next point of increase (passage 13; these passages were defined only for the MSCs used in this study) was correlated with the timing of the dramatic increase in cytokines during cellular senescence (Fig. 1a), indicating that the secretion of cytokines promoted senescence development. During senescence, the cells became glycolytic and shifted to the PPP, as evidenced by increases in glucose-6-phosphate dehydrogenase (G6PDH), NADPH, and total glutathione (GSH) levels (Fig. 1c; Supplementary Fig. 1a–c), which is consistent with a previous report^32^. However, a decrease in mitochondrial function was observed (Fig. 1d). These results revealed a possible functional link between the glycolytic state and the establishment of cellular senescence, implying that aging-associated mitochondrial degenerative changes lead to a shift to a glycolytic transition in senescent cells.

Considering that metabolic rewiring to the glycolytic state contributes to the SASP^33,34^, we confirmed that TIGAR gradually increased during the senescence process of MSCs (Fig. 1e, f; Supplementary Fig. 1d). To examine whether the increase in TIGAR affects SA phenotypes, we knocked out TIGAR via the CRISPR/CAS9 system in cells passaged before the intermediate stage (P7), which corresponds to periods of senescence showing a log phase of SA β-gal activity. Compared with non-KO control cells, both TIGAR knockout (KO) MSCs presented a relative decrease in SA β-gal activity at P5 (2.0-, 1.74-, and 1.60-fold increases in the control, TIGAR KO#1, and TIGAR KO#2, respectively; Fig. 1g). TIGAR KO reduced the secretion of the cytokines interleukin (IL)-6 and MCP-1, which are involved in cell senescence (Fig. 1h; Supplementary Fig. 2a), and decreased the levels of a wide spectrum of secretory factors from senescent cells, including the extracellular matrix, immune modulators, and growth factors (Supplementary Table 3). On the other hand, TIGAR KO-MSCs presented a lower cumulative population doubling (PD) curve than non-KO control cells did and increased expression of markers of cell cycle arrest (Supplementary Fig. 2b; Fig. 1i), indicating that TIGAR KO led to early senescence initiation but delayed senescence progression in MSCs.

### TIGAR KO induces an alteration in the lysosome positioning and a decrease in the autophagic flux

**Fig. 2:**
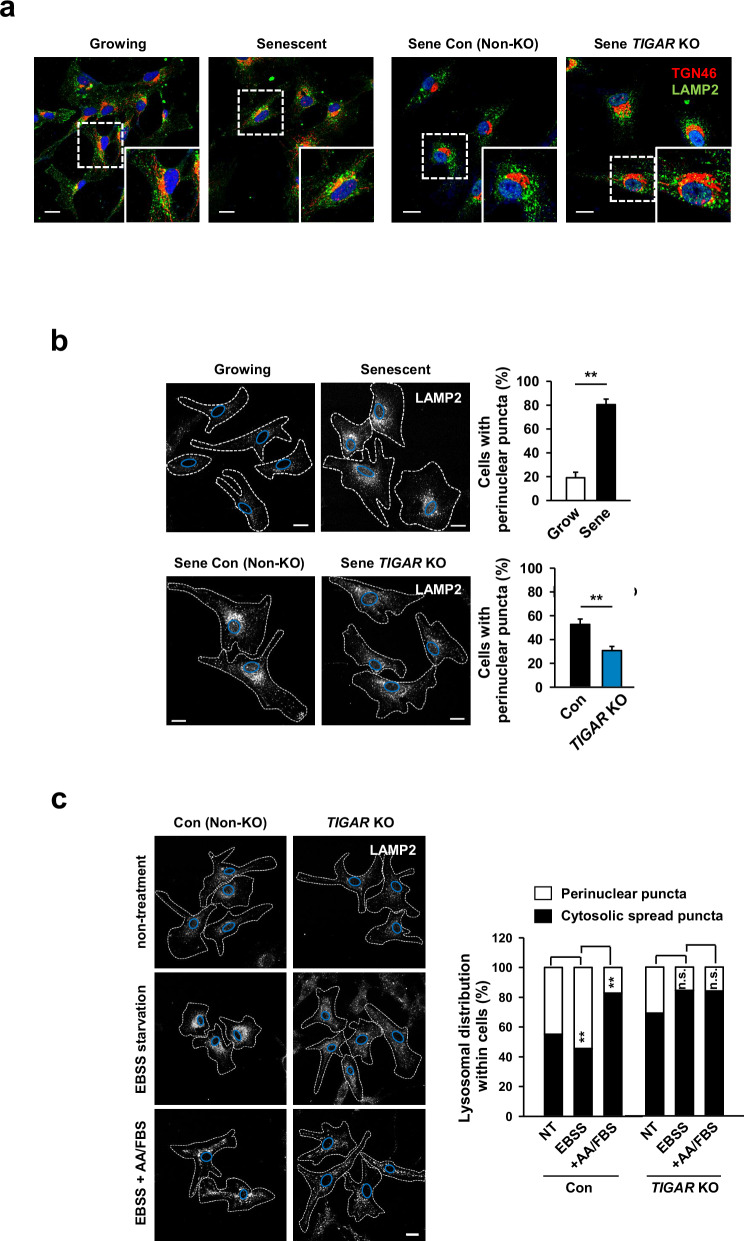
TIGAR KO impairs the spatial association of lysosomes and inhibits the lysosomal positioning to the perinuclear region. **a** Confocal images of TGN46 and LAMP2 immunofluorescence in native MSCs showing growing or senescent status (growing, P7; senescent, P14) and in senescent TIGAR KO-MSCs (P15). TGN46, Trans-Golgi network marker. A magnified image of dotted boxes was inserted. Scale bars: 20 μm. **b** Representative images of the subcellular distribution of lysosomes in native growing and senescent MSCs (P8 and P15, upper) and in senescent TIGAR KO-MSCs (P16, bottom). Cells were immunostained by the LAMP2 antibody. The percentage of cells with predominantly perinuclear localization of lysosomes was quantitatively analyzed and displayed on the right (at least 20 cells per condition). Scale bars: 20 μm. **c** Lysosomal positioning in response to starvation and nutrient addition. Cells were left untreated, starved in EBSS for 50 min, or starved and then recovered in nonessential amino acids (AA)/FBS (1%)-containing medium for 30 min, and immunostained by the LAMP2 antibody. Lysosomal distribution in each experimental condition was quantitatively analyzed (at least 30 cells per condition). Scale bars: 20 μm. White dotted lines in images of (**b**) and (**c**) outline the cell boundaries and blue lines indicate the nuclear shape. Data are means ± SDs from three biological replicates (**b** and **c**). n.s.: not significant; **p < 0.01.

A few reports have shown that senescent cells exhibit a distinct cytoplasmic area where lysosome-associated mTOR accumulates, called the TOR-autophagy spatial-coupling compartment (TASCC)^14,31^. Accordingly, the trans-Golgi network was located near the TASCC in senescent cells. Although we observed secretory phenotypes in native MSCs during senescence, senescent TIGAR KO-MSCs presented no signs of TASCC and retained a peculiar pattern of the Golgi apparatus similar to that in native growing cells (Fig. 2a).

To investigate the cause of spatial disorganization in TIGAR KO-MSCs, we first examined changes in lysosomal movement in native MSCs. In growing MSCs, lysosomes are scattered throughout the cytoplasm, but during the senescence process, they congregate around the nucleus, increasing the number of lysosomal puncta (Fig. 2b, upper). Interestingly, TIGAR KO cells displayed prominent lysosomal puncta throughout the cytoplasm despite their senescence status (Fig. 2b, bottom). Nutrient adjustment in TIGAR KO cells did not affect the redistribution of lysosomes (Fig. 2c). In contrast, control cells presented increased numbers of lysosomal puncta in the perinuclear region after starvation, and the lysosomes were moved to the cytoplasm in response to subsequent nutrient addition.

**Fig. 3:**
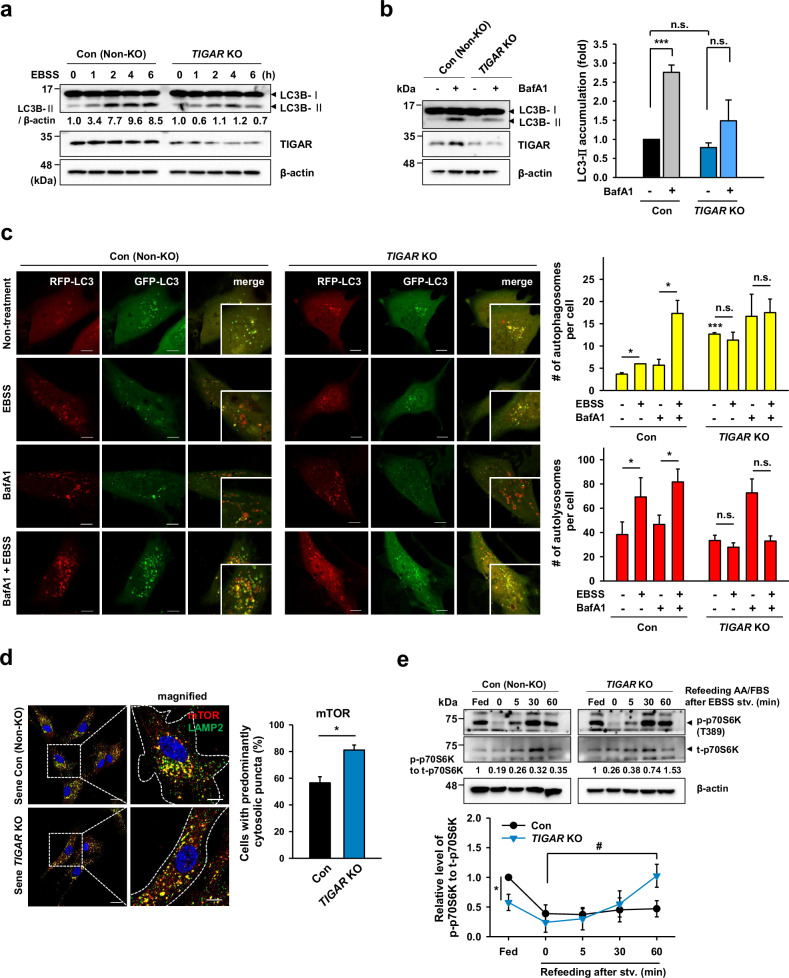
TIGAR KO reduces the autophagic flux and induces diffused mTOR distribution and mTORC1 signal activation. **a**, **b** MSCs starved with EBSS for times indicated and treated with or without the autophagy inhibitor, bafilomycin A1 (10 nM, 4 h), were subjected to immunoblotting. The relative level of LC3-II to β-actin was compared as an indicator of autophagic flux and represented as LC3-II accumulation in the *y*-axis (**c**). MSCs transfected with the mRFP-EGFP/MAP1LC3B plasmid were subjected to EBSS starvation for 1 h after bafilomycin A1 treatment (20 nM, 1 h). Representative fluorescence images were shown on the left. In green- and red-merged images, autophagosomes and autolysosomes are indicated as yellow (GFP^+^/mRFP^+^) and red (GFP^−^/mRFP^+^) puncta, respectively. The number of autophagosomes and autolysosomes was counted (at least 20 cells per condition). Magnified areas of the merged images were inserted. Scale bars: 10 μm. **d** Confocal images of mTOR and LAMP2 immunofluorescence in senescent TIGAR KO-MSCs (P15). The percentage of cells with predominantly cytosolic localization of mTOR was quantitatively analyzed (at least 10 cells). White dotted lines in magnified images outline the cell boundaries. Scale bars: 10 μm. **e** Cells were starved in EBSS for 30 min and then refed with nonessential amino acids (AA)/FBS (1%) for the indicated periods. Cell lysates were subjected to immunoblotting with phosphorylated p70S6K (T389) and total p70S6K antibodies. The ratio of p-p70S6K to t-p70S6K was quantitatively analyzed, and the relative values were displayed below the blots. Graphed data was represented as the value relative to control (non-KO) cells. ^#^ indicates statistical significance in the TIGAR KO cells. Data are means ± SDs from three biological replicates. n.s.: not significant; *p < 0.05, ***p < 0.005.

As the positioning of lysosomes affects their functional phenotypes, we verified autophagic flux by assessing the levels of the LC3-II protein and the fluorescence signals of the mRFP-GFP/LC3 reporter, in which a yellow signal (from green and red) is active in autophagosomes and a red signal is prominent in autolysosomes^27^ following starvation and either in the presence or absence of BafA1. TIGAR KO-MSCs presented relatively late and low LC3B-II expression levels, whereas non-KO control cells presented early and protracted responses to starvation (Fig. 3a). Additionally, TIGAR KO-MSCs presented a low basal flux, as evidenced by the accumulation of the LC3-II protein in the presence of BafA1 (67% of the control, Fig. 3b). The mRFP-GFP/LC3 results revealed that TIGAR KO cells did not increase the proportion of autophagosomes or autolysosomes upon autophagy induction (Fig. 3c). These results indicated a lack of lysosomes located in the perinuclear region for autophagy activation. Furthermore, with respect to subcellular mTOR distribution and signal activation, TIGAR KO cells exhibited the diffusion of mTOR to the periphery (Fig. 3d) without a change in the colocalization of mTOR and lysosomes. In contrast, in control senescent cells, mTOR congregated in distinct perinuclear areas, showing a spatial association with lysosomes. Additionally, TIGAR KO-MSCs exhibited hyperactive mTORC1 signaling upon refeeding following nutrient deprivation (Fig. 3e). These observations indicate that TIGAR expression modulates mTOR activity by altering lysosomal distribution, which is consistent with a previous report^35^. Taken together, considering that lysosomal positioning to the perinuclear region is important for secretory phenotypes, our results showed that TIGAR involves a subcellular distribution of lysosomes, affecting the secretome and senescence development.

### Peripheral lysosome positioning in TIGAR KO-MSCs is associated with the acetylation of α-tubulin

**Fig. 4:**
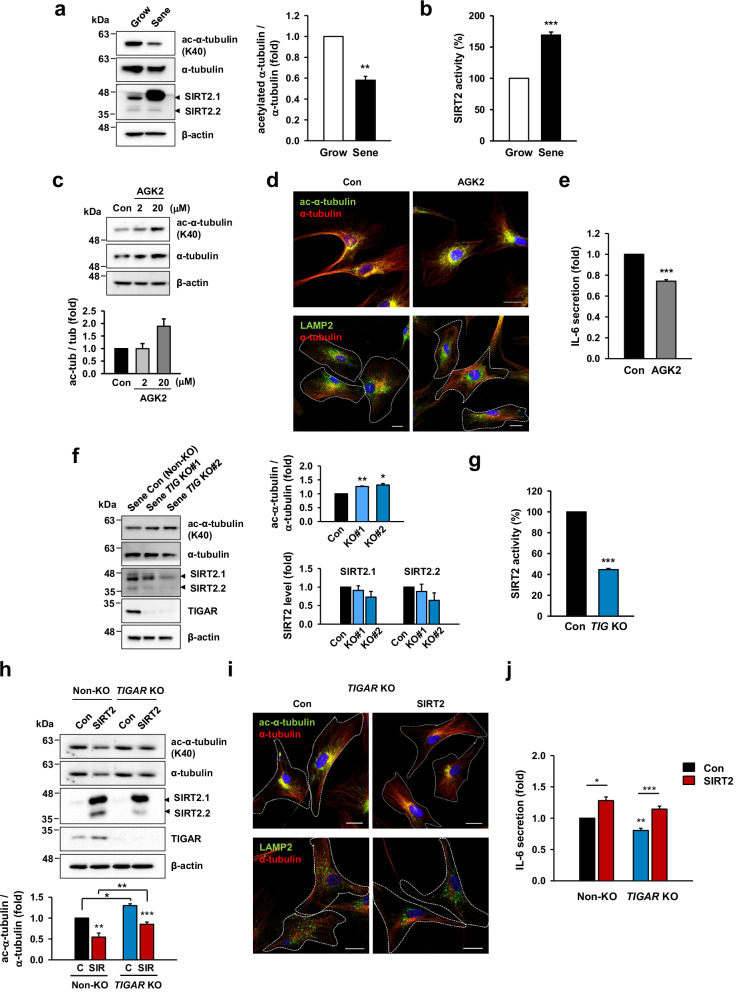
TIGAR influences the acetylation of α-tubulin and lysosomal positioning by regulating SIRT2 activity. **a** Western blot analysis of α-tubulin acetylation and SIRT2 expression in native growing and senescent MSCs (P8 and P16) and the quantitative analysis of α-tubulin acetylation. **b** SIRT2 activity in growing and senescent MSCs. SIRT2 activity in senescent MSCs was represented as a percentage relative to that in growing cells. **c** MSCs were treated with the SIRT2 inhibitor, AGK2 (2 or 20 μM, 6 h), and western blotting and quantitative analysis were conducted to evaluate α-tubulin acetylation. **d**, **e** MSCs treated with AGK2 (10 μM, 24 h) were immunostained by α-tubulin, acetyl α-tubulin (Lys-40), or LAMP2 antibodies (**d**) and measured for IL-6 secretion (**e**). **f** The levels of acetylated α-tubulin and SIRT2 expression in senescent TIGAR KO-MSCs were quantitatively analyzed using immunoblotting. **g** SIRT2 activity of TIGAR KO-MSCs was represented as the percentage relative to that of control (non-KO) cells. **h** MSCs were transduced with a lentiviral vector expressing SIRT2. The α-tubulin acetylation was analyzed quantitatively using immunoblotting. **i**, **j** TIGAR KO-MSCs overexpressing SIRT2 were immunostained by α-tubulin, acetyl α-tubulin (Lys-40), or LAMP2 antibodies (**i**) and were measured for IL-6 secretion (**j**). White dotted lines in (**d**) (bottom) and (**i**) outline the cell boundaries. Scale bars: 10 μm. Data are means ± SDs from three biological replicates (**a**–**c**, **e**–**h**, **j**). *p < 0.05, **p < 0.01, ***p < 0.005. Representative images of western blots and immunostaining were shown.

Lysosomes move to the cell periphery along acetylated microtubules in the perinuclear regions of the cells^21^. Tubulin acetylation, which occurs on Lys-40 (K40) of α-tubulin, is one of the most important posttranslational modifications of microtubules and is associated with various cellular processes^36,37^. We confirmed that MSCs lacked acetylated α-tubulin during senescence (Fig. 4a) and that compared with growing MSCs, senescent MSCs presented increased SIRT2 activity (Fig. 4b). Additionally, treatment with a SIRT2-specific inhibitor (AGK2) increased the level of acetylated α-tubulin, a protein substrate of SIRT2 (Fig. 4c), and induced hyperacetylation of perinuclear microtubules (Fig. 4d, upper). Moreover, SIRT2 inhibition caused lysosome accumulation near the nucleus (Fig. 4d, bottom) and affected IL-6 secretion (Fig. 4e).

On the other hand, TIGAR KO-MSCs did not show a decrease in acetylated α-tubulin levels during senescence (Fig. 4f), in which the cells presented a decrease in SIRT2 expression and a significant reduction in its activity (Fig. 4f, g). We observed that deacetylated α-tubulin in cells with SIRT2 overexpression was weakened by TIGAR KO (Fig. 4h, i), demonstrating that TIGAR regulates SIRT2 activity-related α-tubulin acetylation. Moreover, SIRT2 overexpression in TIGAR-KO cells induced the rearrangement of lysosomes surrounding the nucleus (Supplementary Fig. 3; Fig. 4i). Although a slight difference was observed, SIRT2 expression improved IL-6 secretion in TIGAR-KO cells compared with that in control cells (1.28-fold and 1.43-fold increases in the control and TIGAR-KO cells, respectively; Fig. 4j). These results demonstrated that TIGAR expression influences lysosome positioning at the perinuclear region and secretome through the SIRT2-mediated deacetylation of microtubules.

### TIGAR affects the acetylation of α-tubulin by regulating SIRT2 activity

**Fig. 5:**
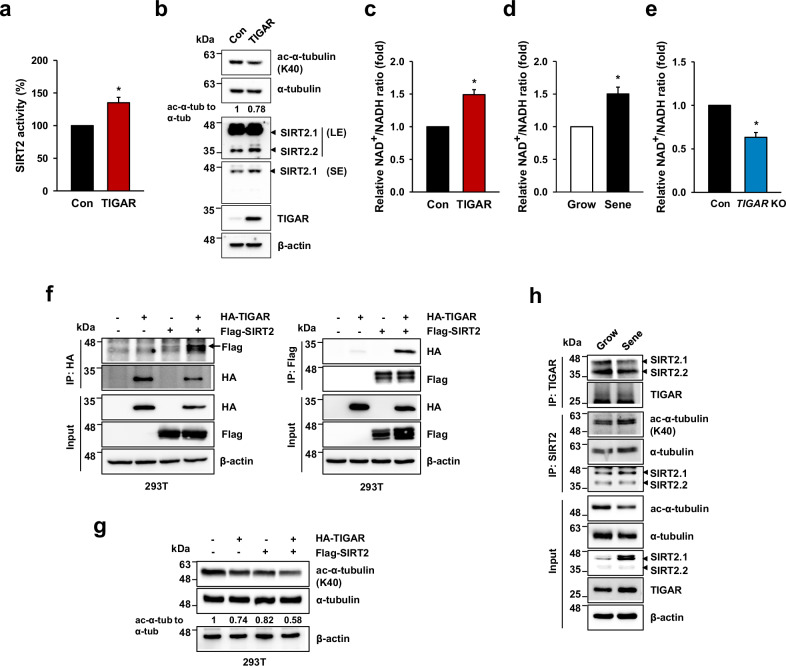
TIGAR regulates α-tubulin acetylation through the interaction with SIRT2. **a**, **b** MSCs were transduced with a lentiviral vector expressing TIGAR. SIRT2 activity was measured (**a**), and the acetylated α-tubulin level was analyzed as a protein substrate of SIRT2 using immunoblotting (**b**). The level of acetyl-α-tubulin to α-tubulin was quantified and shown as the value relative to the control cells below the blots. **c**–**e** The ratio of NAD^+^ to NADH was measured in TIGAR-overexpressed MSCs (**c**) native growing and senescent MSCs (**d**), and TIGAR KO-MSCs (**e**). Data were presented as fold changes relative to the control cells or growing cells, respectively. **f**, **g** 293T cells expressing HA-TIGAR and/or Flag-SIRT2 were immunoprecipitated with anti-HA or anti-Flag antibodies and subjected to western blotting with antibodies against Flag or HA (**f**). Whole-cell lysates were immunoblotted by acetyl-α-tubulin and α-tubulin antibodies. The ratio of acetyl-α-tubulin to α-tubulin was quantitatively compared (**g**). **h** Native growing and senescent MSCs (P7 and P15) were immunoprecipitated with TIGAR or SIRT2 antibodies and subjected to western blotting to observe the interaction of SIRT2 and TIGAR or SIRT2 and α-tubulin. Input cell lysates were immunoblotted by the indicated antibodies. Data are mean ± SD (**a**) or means ± SEs (**c**–**e**) from three biological replicates. *p < 0.05. All immunoblot experiments were shown as representative images of at least three independent blots.

To elucidate the correlation between TIGAR and SIRT2 proteins and the effect on the consequent acetylation of α-tubulin, we investigated SIRT2 activity in TIGAR-overexpressed MSCs. SIRT2 activity significantly increased in TIGAR-overexpressed cells (1.35-fold increase, Fig. 5a), and immunoblotting revealed the deacetylation of Ac-α-tubulin (Fig. 5b). As SIRT2 is an NAD^+^-dependent deacetylase, we investigated whether TIGAR expression causes changes in the ratio of NAD^+^ to NADH. TIGAR-overexpressed MSCs and native senescent MSCs presented an increase in the ratio of NAD^+^ to NADH (1.49-fold and 1.50-fold, respectively; Fig. 5c, d), whereas TIGAR KO-MSCs presented a decrease of 0.63-fold (Fig. 5e), implying that TIGAR expression could regulate SIRT2 activity by regulating the cellular energy status.

Given that the regulation of SIRT2 activity by TIGAR is unclear, we first conducted experiments to verify the interaction of TIGAR with SIRT2 in 293T cells with TIGAR and/or SIRT2 overexpression. When immunoprecipitation assays were performed in the cells, the interaction between TIGAR and SIRT2 was confirmed under reciprocal experimental conditions (Fig. 5f). Subsequent in vitro translation experiments revealed that TIGAR and SIRT2 do not interact directly but rather that these two proteins together interact with other complex proteins (Supplementary Fig. 4). Immunoblotting of cell lysates revealed that the interaction between TIGAR and SIRT2 affected α-tubulin acetylation (Fig. 5g). Furthermore, we observed both interactions in native growing MSCs and verified the increase in the binding of SIRT2 to α-tubulin and the consequential deacetylation of α-tubulin in senescent MSCs, although a slight decrease in the interaction of TIGAR and SIRT2 with senescence was observed (Fig. 5h). These results demonstrated that TIGAR regulates the deacetylation of Ac-α-tubulin through SIRT2 activation; however, further mechanistic studies are needed to identify a functional association between TIGAR and SIRT2.

### TIGAR KO-MSC secretome affects macrophage activity

**Fig. 6:**
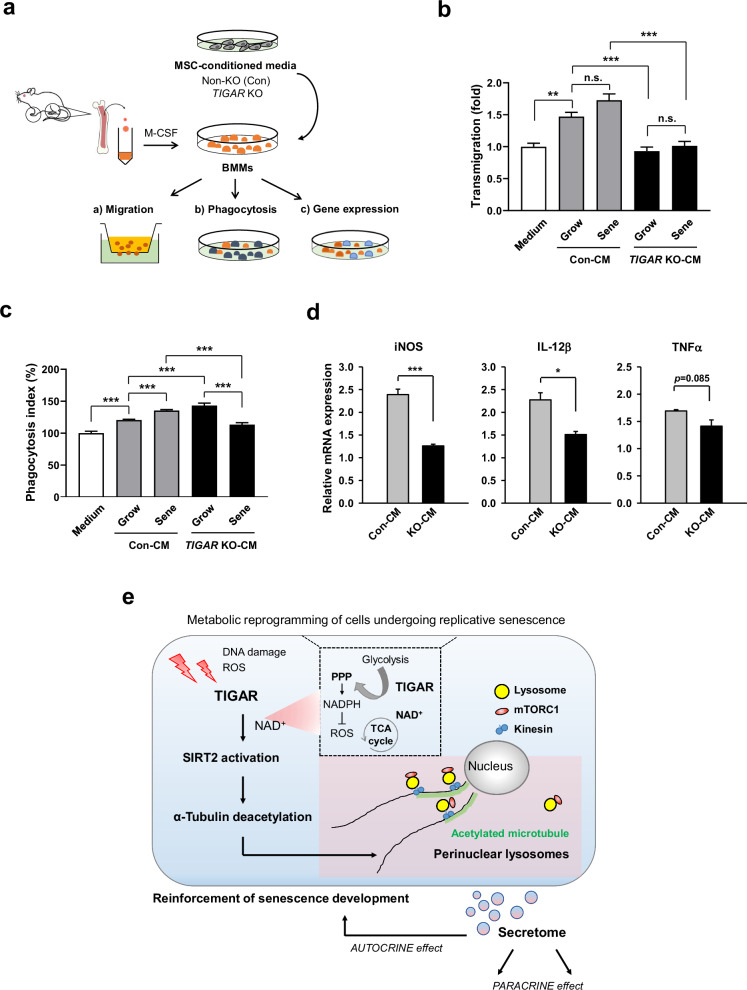
TIGAR KO-MSC secretome decreases the migration and phagocytosis of macrophages. **a** Schematic showing bone marrow-derived macrophages (BMMs) from mice were cultured in an MSC-conditioned medium (MSC-CM). **b**, **c** Non-KO (control)-MSCs and TIGAR KO-MSCs showing growing or senescent status were incubated in a growth medium with 3% FBS for 48 h. BMMs were exposed to the conditioned medium (CM) prepared from the control or TIGAR KO-MSCs for 24 h. Chemotactic potential (**b**) and phagocytosis of BMMs (**c**) were assessed by a migration assay using the Transwell system and by phagocytized FITC-labeled zymosan particles, respectively. The number of cells that migrated through the Matrigel matrix was quantified (**b**). Cells positive for FITC uptake were represented as the phagocytosis index of BMMs (**c**). Data were represented as a value relative to that of the growth medium without cells. **d** The mRNA expression levels of iNOS, IL-12β, and TNFα were analyzed in BMMs treated with MSC-CM for 24 h. Data were expressed as fold changes in senescent cells relative to growing cells (control and TIGAR KO-MSCs). **e** Schematic diagram illustrating SIRT2-mediated acetylation of α-tubulin, lysosome positioning, and secretome occurring in MSCs undergoing replicative senescence. Data are means ± SDs from three biological replicates (**b**–**d**). n.s.: not significant; *p < 0.05, **p < 0.01, ***p < 0.005.

The secretome obtained from cultured MSCs predicts the paracrine effects in the characteristic microenvironment^38,39^. To further assess the impact of TIGAR on SASP secretion, we evaluated the proinflammatory secretome profile of TIGAR-expressing and TIGAR KO-MSCs using bone marrow-derived macrophages (BMMs) stimulation (Fig. 6a). As expected, treatment with senescent MSC-CM enhanced the migration of BMMs and their phagocytosis compared with that of growing cells (Fig. 6b, c). Compared with senescence alone, treatment with TIGAR KO-MSC-CM significantly reduced the transmigration and phagocytosis of BMMs, especially in the context of senescence. Analysis of the mRNA expression of CM-exposed macrophages revealed that TIGAR KO did not induce an increase in macrophage phenotypes, as observed in control senescent cells (Fig. 6d), indicating that TIGAR expression affects the paracrine ability of MSCs to modulate macrophage activity.

In this study, we demonstrated that MSCs increase TIGAR expression during replicative senescence and that TIGAR is involved in lysosomal movement by regulating α-tubulin acetylation through SIRT2 activation, contributing to senescence-associated phenotypes such as the secretome and autophagy (Fig. 6e).

## Discussion

Human MSCs are the standard choice for cell therapy because of the various inflammatory factors that they secrete^38–40^. However, owing to the potential side effects of MSC-based treatments, the MSC-secretome has garnered increasing interest as an alternative and ideal cell-free therapeutic option. A wide spectrum of MSC-secretome-related biological activities, including anti-inflammatory, antiapoptotic, and immunomodulatory effects, has been identified. These findings emphasize the importance of identifying key senescent phenotypes influencing the secretome and understanding the regulatory mechanisms involved. This study revealed that TIGAR influences the senescence-associated MSC-secretome by regulating lysosomal movement.

TIGAR, a glycolytic regulator, is known to regulate redox homeostasis and provide the intermediates necessary for cell growth to protect cells in response to various stressors^41–43^. However, the association of TIGAR with senescence remains confounded across cell types and contexts^44,45^. By performing two genetic manipulation experiments, we observed an increase in TIGAR expression during long-term in vitro culture of MSCs and confirmed the feasibility of increasing TIGAR expression in the context of cellular senescence, whereas TIGAR KO caused early onset but slow progression of senescence (Supplementary Fig. 2b). TIGAR overexpression improved cell proliferation and delayed the onset of senescence (Supplementary Fig. 5a–c). These results led us to hypothesize that TIGAR has compensatory and/or unrevealed functions in senescence processes.

Many reports have shown that TIGAR inhibits autophagy under various pathological conditions^7,46^, which can be explained by the modulation of ROS levels^47,48^ or by changes in the levels of glycolytic intermediates such as glyceraldehyde-3-phosphate dehydrogenase (GAPDH), G6PD, and GSH^49–51^. However, contrasting results have been reported in severe renal ischemia/reperfusion injury^48^. We observed that TIGAR KO reduced the basal autophagic flux of MSCs without distinct changes in LC3 levels or autophagy pathway-related protein levels. Subsequent findings demonstrated that the low autophagic flux was associated with the distribution of lysosomes spread in the cytosol of TIGAR KO-MSCs, indicating a lack of perinuclear lysosomes for autophagosome maturation. In line with this observation, TIGAR KO altered the location of lysosome-bound mTOR to the periphery and induced hyperactive mTOR signaling with nutrient addition. Furthermore, the impact of TIGAR on lysosome positioning and mTOR activation revealed the importance of TIGAR expression for efficient autophagy.

Lysosomes play an important role as metabolic signaling platforms in establishing cellular senescence^52–54^. The elevated lysosomal content in senescent cells may indicate an attempt to produce more lysosomes by counterbalancing the accumulation of dysfunctional lysosomes during cell senescence^55,56^. This balance is maintained during oncogene-induced senescence (OIS) through the TOR–autophagy spatial-coupling compartment^53^. Additionally, our study demonstrates the importance of lysosome repositioning during cellular senescence and the associated regulatory mechanisms. Furthermore, for lysosomal movement along microtubule tracks, acetylation at Lys-40 (K40) of α-tubulin modulates the regional control of lysosome movement^36^ and acts as a guidance cue for enhancing the recruitment of the kinesin-1 (KIF5) motor on microtubules^57^. Whereas senescent MSCs presented a decrease in acetylated α-tubulin levels (Fig. 4a) with a reduction in the KIF5B protein level (Supplementary Fig. 6), senescent TIGAR KO-MSCs presented an increase in acetylated α-tubulin (Fig. 4f) without a decrease in the KIF5B level (Supplementary Fig. 6), which likely resulted in a lysosomal distribution to the periphery of TIGAR KO-MSCs.

To understand the upstream signaling pathway affecting the level of acetylated α-tubulin in MSCs expressing TIGAR, we focused on the activity of the tubulin deacetylase SIRT2, as we verified that in native senescent MSCs, hypoacetylation of α-tubulin was correlated with high SIRT2 activity, which is consistent with previous results^24,58^. As we expected, the acetylation level of α-tubulin in TIGAR KO cells was attributed to relatively low SIRT2 activity compared with that in non-KO control cells. The results of SIRT2 overexpression indicated that SIRT2-mediated deacetylation of α-tubulin enables the flexible movement of lysosomes in the perinuclear region and consequently induces secretory phenotypes. Taken for granted, the deacetylation of Ac-α-tubulin can be modulated by another deacetylase, HDAC6, in apparently redundant reactions with SIRT2. However, based on the HDAC6 activity and inhibitor experiments (Supplementary Fig. 7a–c), we confirmed that HDAC6 globally influences α-tubulin acetylation through cells regardless of TIGAR expression and that SIRT2 may be a critical modulator of α-tubulin acetylation around perinuclear regions. Our idea was supported by previous reports demonstrating differential preferences of the two deacetylases for the recognition of specific structural contexts^59,60^; SIRT2 inactivation was limited to a subset of hyperacetylated perinuclear microtubules inaccessible to HDAC6. However, HDAC6 may affect lysosome movement, as histone acetylation is linked to certain intracellular metabolic or physical cues, including other acetylases and deacetylases.

Furthermore, the upregulation of SIRT2 during cellular senescence is an effect of senescence-associated changes in certain cell types and at least in cells with wild-type p53 status, not a cause of senescence^61^. The enzymatic activity of SIRT2 deacetylase was reported to be linked to the cellular NAD level during aging^62^. However, although we confirmed an increase in SIRT2 activity with cell senescence in human umbilical cord-derived MSCs (UC-MSCs), the increase in SIRT2 activity in senescent MSCs from the intracellular NAD level may be limited by the heterogeneous and disrupted characteristics of senescent cells. Thus, we suggest that SIRT2 activity is closely associated with increased TIGAR expression and changes in the cellular redox balance during senescence.

This study demonstrates the important role of TIGAR in regulating SASP induction and aging development and provides insight into the changes in SIRT2-mediated α-tubulin acetylation and lysosome positioning. Therefore, the reproducibility of our findings in other model systems may contribute to the development of therapeutic strategies for age-related disorders and cancer, thereby reducing the detrimental effects of the SASP while maintaining senescence.

## Supplementary information

Supplementary information

## Data Availability

The data will be made available upon request.
