# Supplementary material for: TIGAR coordinates senescence-associated secretory phenotype via lysosome repositioning and α-tubulin deacetylation
Source: Exp Mol Med. 2024 Dec 4;56(12):2726–38. doi: 10.1038/s12276-024-01362-4 (PMC11671532; doi:10.1038/s12276-024-01362-4)
Supplement: Supplementary file 1 — Supplementary information [file 12276_2024_1362_MOESM1_ESM.pdf]

Supplementary information

TIGAR coordinates senescence-associated secretory phenotype via lysosome repositioning and α-tubulin deacetylation

Nam, H. Y., Park, S.-H., Lee, G.-H., Kim, E.-Y., Lee, S. E., Chang, H. W., Chang, E.-J., Choi, K.-C., and Kim, S. W.

Supplementary Fig. 1 related to Fig. 1

MSCs undergo metabolic rewiring to the glycolytic pathway and increase TIGAR expression during replicative senescence.

Supplementary Fig. 2 related to Fig. 1

TIGAR KO decreases MCP-1 secretion and declines proliferation of MSCs.

Supplementary Fig. 3 related to Fig. 4

SIRT2 overexpression increases lysosomes in the perinuclear region of MSCs.

Supplementary Fig. 4 related to Fig. 5

TIGAR and SIRT2 do not interact directly in MSCs.

Supplementary Fig. 5 related to Fig. 1

TIGAR overexpression improves proliferation of MSCs, and induces a decrease of cytokine secretion and a delayed onset of cellular senescence.

Supplementary Fig. 6

TIGAR KO-MSCs do not show an increase of KIF5B expression during cellular senescence.

Supplementary Fig. 7

HDAC6 influences acetylation of α-tubulin regardless of TIGAR expression.

Supplementary Table 1

List of primers used for qPCR

Supplementary Table 2

List of antibodies

Supplementary Table 3

Comparison of secretome profiling between the control and TIGAR KO-MSCs in the senescent status

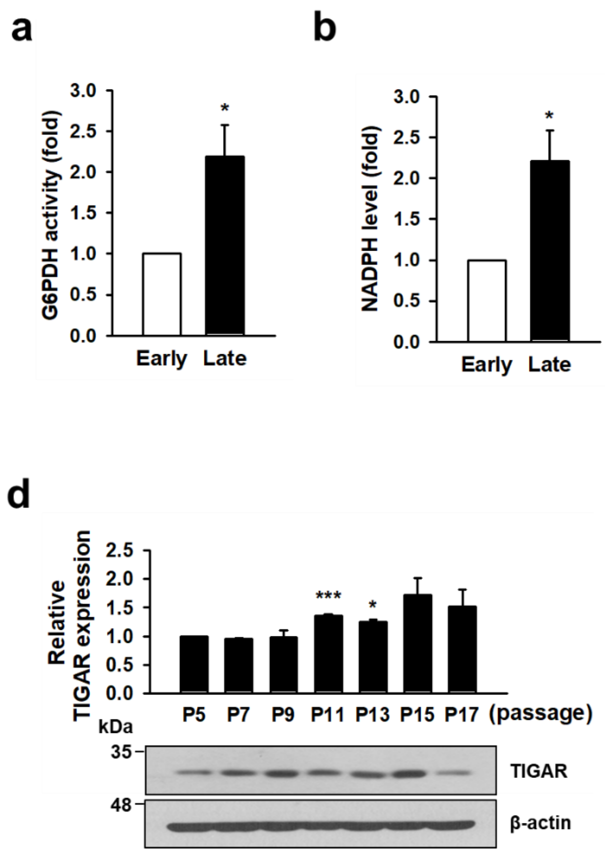

Supplementary Fig. 1 MSCs undergo metabolic rewiring to the glycolytic pathway and increase TIGAR expression during replicative senescence. UCB-MSCs were maintained to lose their proliferative capacity during the *in vitro* expansion. Senescence process of MSCs was classified into three stages of early (P5 to P7), intermediate (P8 to P12), and late (P13 to P17; see Fig. 1**a**). **a**–**c** The G6PDH activity (**a**), NADPH level (**b**), and total GSH level (**c**) were measured in MSCs in early and late stages of replicative senescence. **d** The expression level of TIGAR protein and the quantitative analysis were conducted following *in vitro* cell passaging. TIGAR was normalized to β-actin. Data are mean ± SD from at least three biological replicates. n.s.: not significant; $^{*}p < 0.05$, $^{***}p < 0.005$. Representative images of three independent blots were shown in **d**.

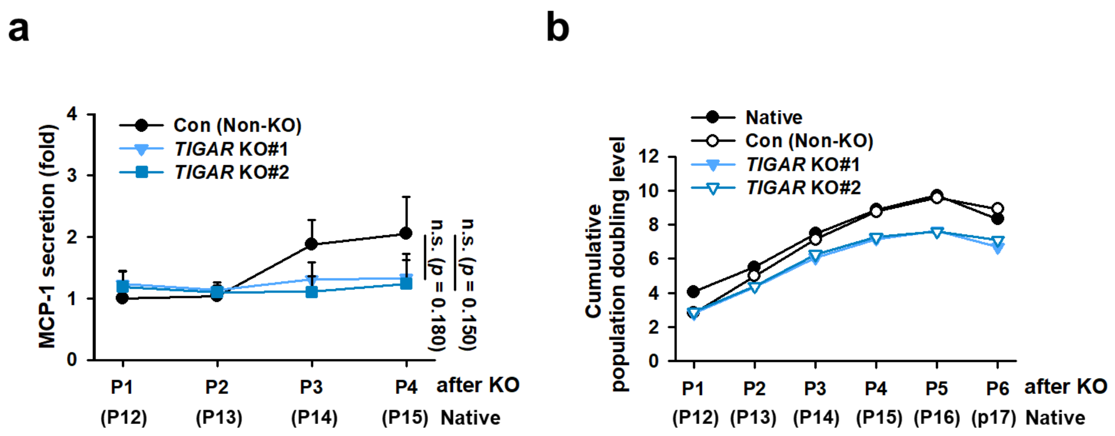

Supplementary Fig. 2 TIGAR KO decreases MCP-1 secretion and declines proliferation of MSCs. a, b MSCs were knocked out TIGAR using the CRISPR/CAS9 system for two different guide RNAs in the cells of passage before the intermediate stage (P7). MCP-1 secretion (**a**) and cumulative population doubling (**b**) were measured over passaging after knocking out TIGAR. Data on cytokine was normalized by cell number (1×10^4 cells) and represented as fold changes relative to the initial passage of control cells (n = 4). Passages after KO (P1 to P4 or to P6) in the x-axis of **a** and **b** correspond to passages 12 to 17 (or 15) of native control cells. Data are mean ± SD (**a**) or mean (**b**) from at least three biological replicates. n.s.: not significant.

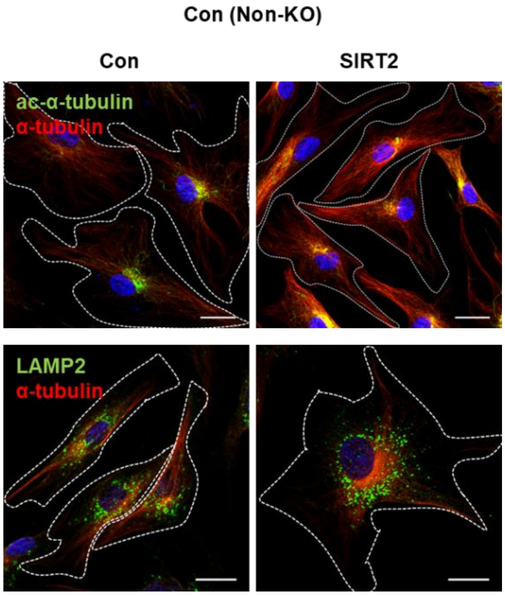

Supplementary Fig. 3 SIRT2 overexpression increases lysosomes in the perinuclear region of MSCs. SIRT2 overexpression was achieved by lentiviral infection in control (non-KO) MSCs. After 48 h, cells were immunostained with α-tubulin, acetyl α-tubulin (Lys40), or LAMP2 antibodies. Representative images of immunostaining were shown. White dotted lines in images outline the cell boundaries.

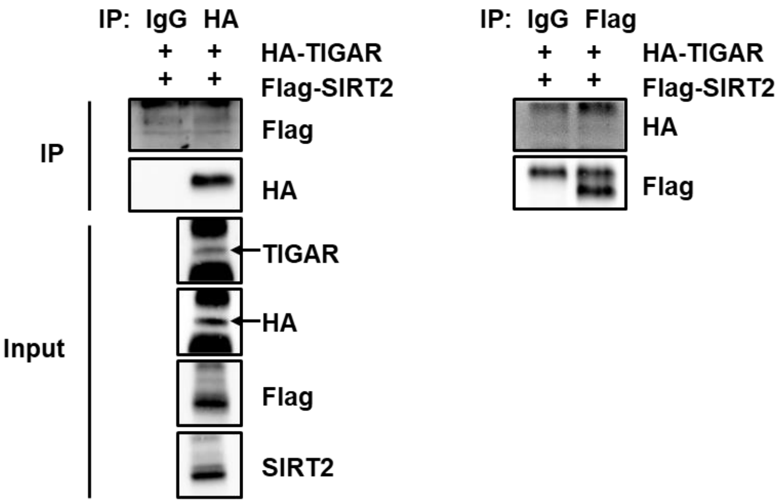

Supplementary Fig. 4 TIGAR and SIRT2 do not interact directly in MSCs. HA-TIGAR and Flag-SIRT2 were translated in vitro using the TNT® Quick Coupled Transcription/ Translation System. The proteins were immunoprecipitated with either HA, Flag, or IgG antibodies and detected by western blot with indicated antibodies.

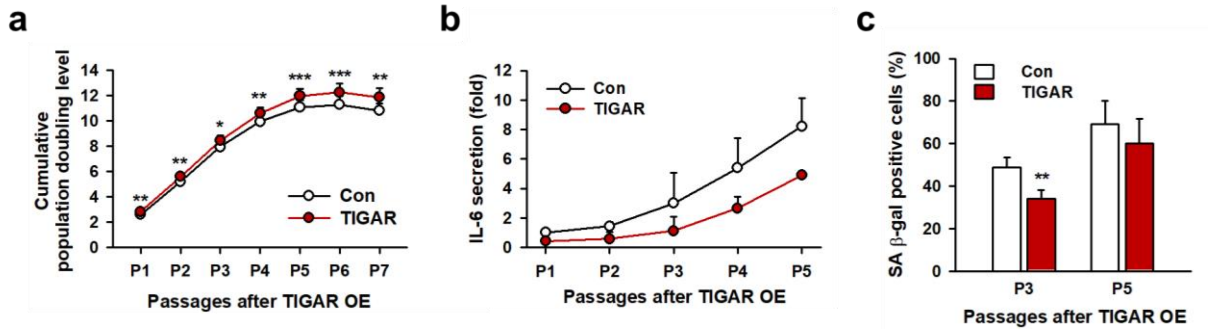

Supplementary Fig. 5 TIGAR overexpression improves proliferation of MSCs, and induces a decrease of cytokine secretion and a delayed onset of cellular senescence. a–c MSCs were transduced with a lentiviral vector expressing TIGAR. Cumulative population doubling (**a**), IL-6 secretion (**b**), and the percentage of cells with positive SA β-gal staining (**c**) were measured over passaging after overexpressing TIGAR. Data on cytokine was normalized by cell number (1×10^4 cells) and represented as fold change relative to the initial passage of control cells (n = 4). Passages after overexpression (P1 to P7) correspond to passages 9 to 15 of native control cells. Data are mean ± SD from three to four biological replicates. $^{*}p < 0.05$, $^{**}p < 0.01$, $^{***}p < 0.005$.

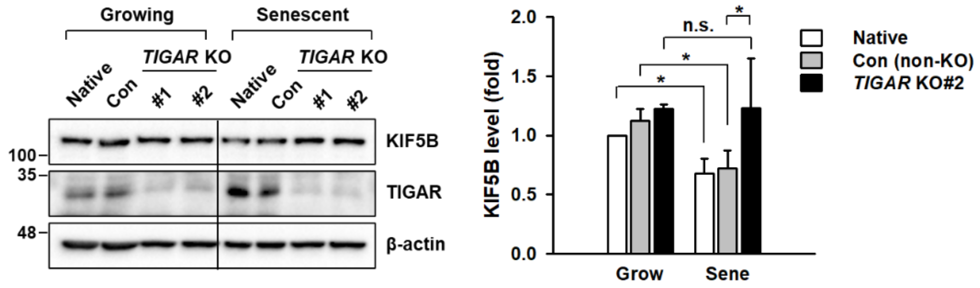

Supplementary Fig. 6 TIGAR KO-MSCs do not show an increase of KIF5B expression during cellular senescence. The levels of KIF5B expression were measured in growing- and senescent-native MSCs and TIGAR KO-MSCs (growing, P10; senescent, P15). To identify changes in KIF5B expression during MSC senescence, the level was quantitatively analyzed and compared with the native growing MSCs. Data are mean ± SD from three biological replicates. n.s.: not significant; * $p < 0.05$.

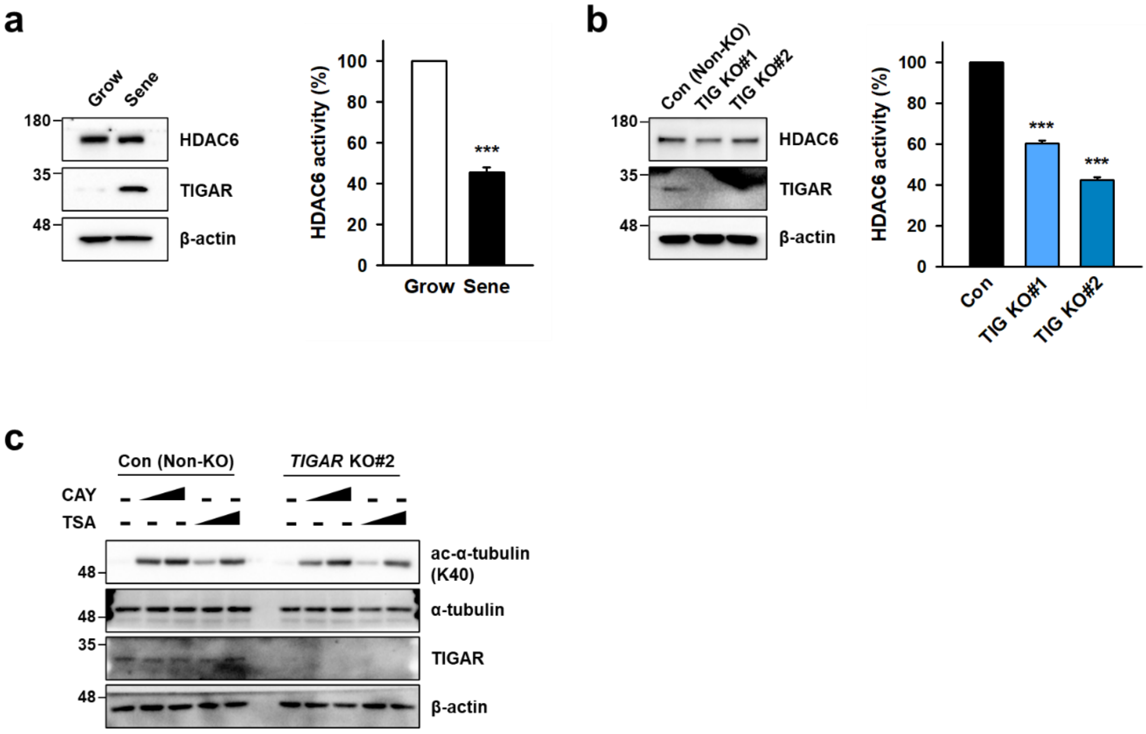

Supplementary Fig. 7 HDAC6 influences acetylation of α-tubulin regardless of TIGAR expression. a, **b** Western blot analysis of HDAC6 expression and the activity were measured in native growing and senescent MSCs (**a**, P8 and P15) and TIGAR KO-MSCs (**b**, P13). HDAC6 activity was represented as percentage relative to that in growing cells and non-KO cells, respectively. **c** Cells were treated with the HDAC6 inhibitors, CAY10603 (1, 5 μM, 24 h) and Tubastatin A (TSA; 1, 10 μM, 24 h), and the levels of acetylated α-tubulin were analyzed by western blotting. Data (**a** and **b**) are mean ± SD from three biological replicates. $^{***}p < 0.005$. Representative images of blots were shown.

Supplementary Table 1 List of primers used for qPCR

mRNA	Forward (5'-3')	Reverse (5'-3')
human *TIGAR*	GTATTCCAGGATTAGCAGCCAGTG	GGTTCATACAAATACACTGAACCG
human *GAPDH*	TGAACGGGAAGCTCACTGG	TCCACCACCCTGTTGCTGTA
mouse *iNOS*	AAGCCCCGCTACTACTCCAT	AGCTGGAAGCCACTGACACT
mouse *IL-12β*	TGGTTTGCCATCGTTTTGCTG	ACAGGTGAGGTTCACTGTTCT
mouse *TNFα*	CCCTCACACTCAGATCATCTTCT	GCTACGACGTGGGCTACA
mouse *GAPDH*	TGCAGTGGCAAAGTGGAGATTGTTG	TGTAGCCCAAGATGCCCTTCA G

Supplementary Table 2 List of antibodies

Epitope	Species reactivity	Catalog No.	Company	Use	Dilution	Source
Primary antibodies						
Phospho-Rb (Ser780)	human	9307	Cell Signaling	WB	1:1000	rabbit
Rb	human	9309	Cell Signaling	WB	1:1000	mouse
$p21^{WAF1/Cip1}$	human	2947	Cell Signaling	WB	1:1000	rabbit
$p27^{Kip1}$	human	3688	Cell Signaling	WB	1:1000	rabbit
TIGAR	human	sc-74577	Santa Cruz	WB	1:1000	mouse
LC3B	human	L7543	Sigma-Aldrich	WB	1:1000	rabbit
phospho-p70 S6K (Thr389)	human	9234	Cell Signaling	WB	1:1000	rabbit
p70S6K	human	9202	Cell Signaling	WB	1:1000	rabbit
alpha tubulin (acetyl K40)	human	ab24610	abcam	WB<br>IF	1:1000<br>1:400	mouse
alpha tubulin	human	sc-5286<br>ab52866	Santa Cruz<br>abcam	WB, IP<br>IF	1:1000<br>1:400	mouse<br>rabbit
SIRT2	human	ab211033	abcam	WB, IP	1:1000	rabbit
β-actin	human	A5441	Sigma-Aldrich	WB	1:1000	mouse
LAMP2	human	ab25631	abcam	IF	1:50	mouse
TGN46	human	ab50595	abcam	IF	1:200	rabbit
mTOR	human	2983	Cell Signaling	IF	1:500	rabbit
Flag-tag		F3165	Sigma-Aldrich	IP	1:50	mouse
HA-tag		M180-3	MBL	IP	1:50	mouse
Flag-tag		F7425	Sigma-Aldrich	IP	1:50	rabbit
HA-tag		3724	Cell Signaling	IP	1:50	rabbit
Secondary antibodies						
Anti-mouse HRP	mouse	A90-116P	Bethyl	WB	1:10,000	goat
Anti-rabbit HRP	rabbit	A120-101P	Bethyl	WB	1:10,000	goat
Alexa Fluor™ 488	mouse	A-11029	invitrogen	IF	1:2000	goat
Alexa Fluor™ 555	rabbit	A-21428	invitrogen	IF	1:2000	goat

Supplementary Table 3 Comparison of secretome profiling between the control and TIGAR KO-MSCs in the senescent status

Secretory cytokines		Relative value to Non-Sene (fold)		Fold-change of TIGAR KO to Con
		Sene Con (Non-KO)	Sene TIGAR KO	
TIGAR KO-decreased cytokines	CCL2/JE/MCP-1	4.42	3.30	0.75
	CCL3/MIP-1 alpha	3.74	2.18	0.58*
	CCL4/MIP-1 beta	2.51	1.71	0.68*
	IFN-gamma	2.68	2.13	0.79
	IL-1 alpha/IL-1F1	1.58	1.01	0.64
	IL-17/IL-17A	1.45	1.28	0.88
	IL-18/IL-1F4	4.60	2.17	0.47
	TNF-alpha	1.65	1.16	0.71**
	FGF acidic/FGF1	1.85	1.47	0.79
	HGF #	0.46	0.38	0.83**
	IL-1ra/IL-1F3	2.01	1.57	0.78
	IL-4	2.61	1.65	0.63*
	IL-15	2.49	1.70	0.68*
	IL-23	1.07	0.83	0.77
	CXCL9/MIG	2.02	1.71	0.84
	CXCL13/BLC/BCA-1	2.20	1.61	0.73
TIGAR KO-increased or less sensitive cytokines	G-CSF	3.68	19.74	5.37***
	GM-CSF	4.47	7.17	1.60*
	IL-1 beta/IL-1F2 #	0.88	1.07	1.22
	IL-10	2.84	6.57	2.31
	VEGF	1.14	1.24	1.08*
	CXCL10/IP-10/CRG-2	17.96	19.13	1.06
	CXCL5/ENA-78	3.33	3.53	1.06
	Thrombopoietin/Tpo	1.12	1.18	1.05

Conditioned medium derived from control (non-KO) MSCs and TIGAR KO-MCS in the growing (Non-Sene; passage 2 after KO) or senescent stage (Sene; passage 6 after KO) was analyzed using a customized multiplex assay for the measurement of secretory factors. Data were normalized by the cell number (1×10^4 cells). The level of secretome obtained from non-senescent cells was arbitrarily set at 1, and the relative level of senescent cells was represented as a fold change. Data were classified as decreased cytokines and increased or less sensitive cytokines in the TIGAR KO condition. Three replicates were used per sample. # indicates decreased cytokines in senescent control cells; * indicates significant changes in TIGAR KO cells compared with that in the control cells (*P < 0.05, **P < 0.01, ***P < 0.005).
